# The prevalence of Type D personality and its association with clinical outcomes in patients with cervical radiculopathy after either conservative or surgical treatment

**DOI:** 10.3389/fnhum.2026.1887901

**Published:** 2026-08-20

**Authors:** Azra Gül, Sarita van Geest, Barbara Kuijper, Anton Adriaan Van Der Plas, Carmen Vleggeert-Lankamp

**Affiliations:** 1 Leids Universitair Medisch Centrum Afdeling Neurochirurgie, Leiden, Netherlands; 2 Martini Ziekenhuis, Groningen, Netherlands; 3 Maasstad Ziekenhuis, Rotterdam, Netherlands; 4 Alrijne Ziekenhuis Leiden, Leiden, Netherlands; 5 Spaarne Gasthuis, Haarlem, Netherlands

**Keywords:** ACDF, cervical radiculopathy, conservative, depression, Type D personality

## Abstract

Type D personality, characterized by the combination of negative affectivity and social inhibition, has been associated with adverse outcomes in several patient populations, but its role in patients with cervical radiculopathy remains unknown. This study aimed to evaluate the association between baseline Type D personality and clinical outcomes over a 2 year follow-up period in patients with cervical radiculopathy treated with either conservative or surgical treatment. In addition, we explored baseline characteristics associated with the presence of a Type D personality. Data were derived from the CASINO trial (NTR3504), a prospective multicenter observational cohort study including patients with cervical radiculopathy due to disc herniation. Clinical outcomes included Visual Analog Scale (VAS) arm pain, VAS neck pain, Neck Disability Index (NDI), and EuroQol Visual Analog Scale (EQ-VAS), measured at baseline and during follow-up up to 104 weeks. Linear mixed-effects models were used to evaluate associations between Type D personality and longitudinal clinical outcomes, adjusted for baseline outcome values, age, gender, and BMI. A total of 141 patients were included (mean age 51.1 years; 52% female), of whom 88 (62%) underwent surgical treatment and 40 (28%) had a Type D personality. No significant differences in clinical outcomes were observed during short-term follow-up. Over the 2 year follow-up period, however, in the whole cohort, patients with a Type D personality reported higher VAS arm pain scores compared with patients without a Type D personality (β = 11.72, *p* = 0.030). Similarly, Type D personality was associated with higher NDI scores at 2 years (β = 7.63, *p* = 0.033). Furthermore, patients with a Type D personality reported lower EQ-VAS scores at 1 year, reflecting poorer overall perceived health (β = −10.51, *p* = 0.031). No significant association was observed between Type D personality and VAS neck pain. Baseline characteristics associated with Type D personality included a longer interval between symptom onset and first general practitioner consultation and a higher proportion of patients living alone or not married. Identification of Type D personality may contribute to improved patient counseling and long-term expectation management.

## 1 Introduction

Depression represents a major global public health concern, affecting individuals of all age groups. It is estimated that approximately one in five people will experience depression during their lifetime and it currently ranks among the leading causes of years lived with disability worldwide (Zhdanava et al., 2021; GBD 2023 Disease Injury Risk Factor Collaborators, 2025). The burden of depressive symptoms is particularly pronounced in patients with (sub) chronic pain conditions, where psychological distress frequently coexists with, and may exacerbate, physical symptoms (Aaron et al., 2025; Simon et al., 1996). Psychological vulnerability exists on a continuum and is not limited to individuals with a clinical diagnosis of depression. A personality trait that has gained increasing attention is the Type D (“distressed”) personality, which is characterized by the combined tendency to experience negative emotions (negative affectivity) while simultaneously suppressing the expression of their emotions due to fear of social disapproval or rejection (social inhibition). Individuals scoring high on both personality traits are classified as having a Type D personality (Mols and Denollet, 2010).

Type D personality is estimated to be present in approximately 23-26% of the general population (Hausteiner et al., 2010). Among patients with various spinal disorders, its prevalence has been reported to range from 30-39% (Riediger et al., 2026, 2025; Eroglu et al., 2022). One important subgroup of patients with spinal disorders comprised individuals with cervical radiculopathy. Cervical radiculopathy, most commonly resulting from intervertebral disc herniation, is characterized by radiating arm pain and/or sensory and/or motor deficits due to nerve root compression, and is often accompanied by neck pain. Although the majority of patients experience substantial recovery within the first months after symptom onset, a subset of patients develops persistent symptoms. In such cases, treatment regimens are reconsidered, and a decision is made between prolonged conservative treatment or surgical intervention, depending on symptom severity and shared decision-making. However, clear criteria to guide treatment selection remain lacking, and a substantial proportion of patients report persistent symptoms or dissatisfaction following surgical treatment (Vleggeert-Lankamp et al., 2019).

Several established psychosocial risk factors, including pain catastrophizing, fear-avoidance beliefs, low self-efficacy, and adverse social circumstances, have been associated with pain chronification and impaired functional recovery (Linton, 2000; Vlaeyen and Linton, 2000). Psychological factors may also influence treatment response in patients with cervical radiculopathy. For instance, preoperative depressive symptoms have been associated with less favorable postoperative outcomes, including higher levels of arm and neck pain (Cunningham et al., 2025). Whether Type D personality is similarly associated with treatment outcomes in patients with cervical radiculopathy remains unknown. Furthermore, previous studies assessing the impact of Type D personality in patients with spinal disorders have primarily focused on surgically treated patients, leaving it unclear whether the potential impact of Type D personality differs between patients receiving prolonged conservative treatment and those undergoing surgery. If Type D personality is indeed associated with worse clinical outcomes, early identification of vulnerable patients becomes clinically relevant. Additionally, insight into the baseline characteristics associated with Type D personality may help identify patients at increased risk for unfavorable outcomes.

Therefore, the aim of the present study is to evaluate the association between baseline Type D personality and clinical outcomes in patients with cervical radiculopathy over a 2 year follow-up period, following either conservative or surgical treatment. In addition, we explored the association between Type D personality and a range of patient characteristics including demographic, lifestyle, and disease-related factors. A better understanding of the role of Type D personality in treatment outcomes and patient satisfaction may contribute to improved risk stratification and more personalized treatment strategies for patients suffering from cervical radiculopathy.

## 2 Materials and methods

### 2.1 Study design

Data were derived from the CASINO trial (NTR3504) (Gül et al., 2025), a multi-center observational cohort study including patients with cervical radiculopathy due to cervical disc herniation, comparing conservative vs. surgical treatment strategies. The study was approved by the Medical Ethics Committee of the Leiden University Medical Center, and written informed consent was obtained from all patients prior to enrolment.

### 2.2 Study population

Patients aged 18 to 75 years with cervical radicular syndrome (CRS) due to a herniated disc were considered eligible if they experienced disabling symptoms or tingling sensations for a minimum duration of 2 months. The diagnosis of cervical radiculopathy was established by a neurologist or neurosurgeon at participating centers. Inclusion criteria comprised a Visual Analog Scale (VAS) pain score of ≥40, and MRI confirmation of a herniated disc causing nerve root compression. Exclusion criteria included the presence of arm paresis (MRC grade < 4), signs of myelopathy, prior cervical spine surgery, spinal instability requiring fixation, pregnancy, insufficient proficiency in the Dutch language, planned emigration, or concurrent participation in another clinical trial.

### 2.3 Baseline characteristics

At baseline, patients completed a comprehensive questionnaire capturing demographic, lifestyle, and disease-related characteristics. Demographic variables included age, gender, Body Mass Index (BMI), educational attainment, employment status, and the presence of sick leave. Lifestyle factors comprised alcohol consumption and smoking behavior, as well as engagement in sports or physical activity.

Disease-related characteristics included the patient-reported duration of symptoms, time since the first consultation with a general practitioner for the current complaint, and the relative predominance of arm vs. neck pain. Additionally, patients reported a family history of cervical disc herniation. Medication use was assessed through self-reported use of analgesics, including paracetamol, non-steroidal anti-inflammatory drugs (NSAIDs), tramadol, and/or morphine.

Type D personality was assessed using the Type D Scale-14 (DS-14), a validated questionnaire comprising two subscales: negative affectivity and social inhibition, each consisting of seven items scored on a 5-point Likert scale ranging from 0 to 4 (Denollet, 2005). Subscale scores range from 0 to 28, with higher scores indicating greater levels of negative affectivity or social inhibition. The combined total DS-14 score therefore ranges from 0 to 56, with higher scores reflecting greater overall psychological distress. Negative affectivity reflects the tendency to experience negative emotions, whereas social inhibition refers to the tendency to inhibit self-expression in social interactions due to fear of disapproval. In accordance with established criteria, Type D personality was defined as a score of ≥10 on both subscales (Denollet, 2005).

### 2.4 Clinical outcomes

Arm pain and neck pain were measured using separate Visual Analog Scales (VAS), ranging from 0 to 100 mm, with higher scores indicating greater pain intensity. Neck functionality was assessed using the Neck Disability Index (NDI), a 10-item questionnaire evaluating pain intensity and functional limitations in work-related activity and daily activities, with total scores ranging from 0 (best) to 50 (worst), converted to a 0–100 scale. Health-related quality of life was assessed using the EuroQol Visual Analog Scale (EQ-VAS), ranging from 0 (worst imaginable health) to 100 (best imaginable health). Clinical outcomes were scored at baseline, 6, 12, 26, 38, 52, and 104 weeks of follow-up.

### 2.5 Statistical analysis

Baseline characteristics were reported as mean ± standard deviation (SD) for normally distributed continuous variables and as frequencies with proportions for categorical variables. Mean values ± SD were reported for patients classified as having a Type D personality and for patients not classified as having a Type D personality. In addition, descriptive outcomes were presented separately for conservatively treated and surgically treated patients within both the Type D and non–Type D groups.

Analyses were performed to identify independent associations between Type D personality and 23 distinct demographic, lifestyle, and disease-related characteristics. The association with continuous variables was assessed using independent *t*-tests and for all categorical variables chi-square and/or Fisher's exact tests have been performed (Appendix 1).

Linear mixed-effects models were used in all analyses to account for repeated measurements within subjects and to investigate the association between Type D personality and clinical outcomes on the short-term (6 weeks) and long-term (52 and 104 weeks). All models included Type D personality as a dichotomous fixed effect, together with the baseline value of the respective outcome variable, while subjects were included as random intercepts. The overall effect of Type D personality on VAS arm pain, EQ-VAS, VAS neck pain, and NDI was assessed for the total study population. As NDI data were not collected at the 6-week follow-up, the 12-week assessment was used as the short-term follow-up time point in the adjusted linear mixed-effects models for the NDI only.

Subgroup analyses were additionally performed comparing patients with and without a Type D personality within the conservatively treated group and within the surgically treated group separately.

The models were adjusted for baseline clinical outcomes, age, gender and BMI. A *p*-value < 0.05 was considered statistically significant. All analyses were performed using IBM SPSS Statistics version 30.0.

## 3 Results

### 3.1 Baseline characteristics

A total of 141 patients were included in the analysis, from who 53 received conservative treatment and 88 patients received surgical treatment. The average age of participants was 51.11 years (SD = 9.73), with 52% being female. The mean baseline BMI was 26.51 kg/m2 (SD = 4.15). Having the Type D personality was reported in 40 (28%) patients (Table 1).

**Table 1:** Baseline patient demographics.

All patients (*n* = 141)		
DS-14 (mean ± SD)		19.96 ± 8.25
Type D personality (*n*, %)		40 (28)
Age (years, mean ± SD)		51.11 ± 9.73
Gender (n, %)	Male	67(48)
	Female	74 (52)
Body Mass Index (mean ± SD)		26.51 ± 4.15
Treatment (*n*, %)	Conservative	53 (38)
	Surgical	88 (62)
HNP Location (*n*, %)	C4C5	10 (7)
	C5C6	66 (47)
	C6C7	59 (42)
	C7T1	6 (4)
Daytime Activities (n, %)	1) Housewife/Househusband	9 (6)
	2) Unemployed	19 (13)
	3) Student	0 (0)
	4) Unpaid Work (volunteering)	2 (1)
	5) Part-time work	41 (30)
	6) Full-time work	70 (50)
Education (*n*, %)	1) None/Primary Education	18 (13)
	2) MAVO/VMBO	65 (46)
	3) HAVO/HBS/VWO	15 (11)
	4) HBO/WO (University)	43 (30)
Work activity (*n*, %)	0) Not Applicable	26 (18)
	1) Sitting	47 (33)
	2) Light Physical work	31 (23)
	3) Heavy Physical work	37 (26)
Sick leave (*n*, %)	0) None	61 (43)
	1) Retired/Early Retirement	11 (8)
	2) No paid employment	9 (6)
	3) Disabled	7 (5)
	4) Yes	53 (38)
Alcohol consumption (*n*, %)	0) None	46 (33)
	1) Occasionally	63 (45)
	2) Regularly	32 (23)
Daily Alcohol units (mean ± SD)		1.64 ± 0.73
Smoking behavior (*n*, %)	0) None	88 (62)
	1) Occasionally	8 (6)
	2) Regularly	45 (32)
Number of cigarettes a day (mean ± SD)		12.38 ± 6.98
Married and/or moved in together (*n*, %)		108
Family member with CRS (n, %)	Total	23
	Brother	7
	Dad	10
	Sister	4
	Mum	2
Participation in sports (*n*, %)		75 (53)
Length of complaints (mean months ± SD)		9.85 ± 12.07
Months since first General Practitioner visit (mean months ± SD)		11.23 ± 17.69
Arm pain compared to Neck pain (*n*, %)	1) Arm pain only	11 (8)
	2) Neck pain only	5 (4)
	3) Arm pain equal to neck pain	32 (23)
	4) More arm pain than neck pain	68 (48)
	5) More neck pain than arm pain	25 (18)
Use of paracetamol (n, %)		68 (48)
Use of NSAID's (n, %)		39 (28)
Use of tramadol (n, %)		31 (22)
Use of morphine (n, %)		27 (19)
VAS arm pain (mean ± SD)		56.77 ± 26.80
EQ-VAS (mean ± SD)		52.11 ± 22.51
VAS neck pain (mean ± SD)		52.84 ± 27.15
NDI (mean ± SD)		35.68 ± 14.56

Baseline demographic, lifestyle, disease-related, and clinical characteristics are presented for the total study population (*n* = 141). Continuous variables are reported as mean ± standard deviation (SD), while categorical variables are presented as frequencies with percentages. Clinical outcome measures included VAS arm pain, EQ-VAS, VAS neck pain, and the Neck Disability Index (NDI). Abbreviations: DS-14, Type D Scale-14; HNP, herniated nucleus pulposus; CRS, cervical radicular syndrome; NSAIDs, nonsteroidal anti-inflammatory drugs; NDI, Neck Disability Index; EQ-VAS, EuroQol Visual Analog Scale.

### 3.2 Effects of Type D personality on VAS arm pain

**Figure 1:**
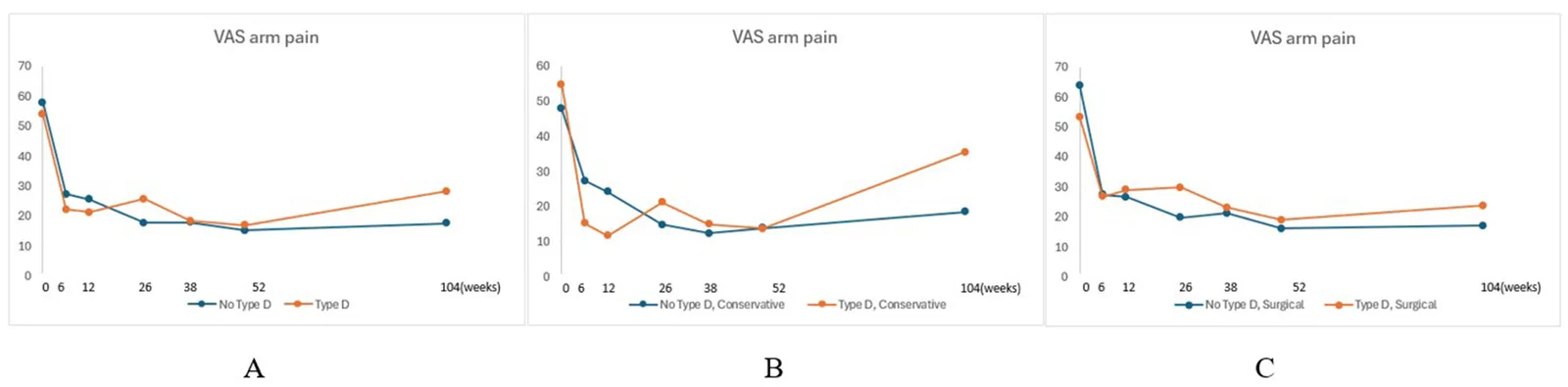
Clinical outcome trajectories for VAS arm pain over time stratified by Type D personality. **(A)** Mean VAS arm pain scores over time for the total study population comparing patients with and without a Type D personality. **(B)** Mean VAS arm pain scores over time among conservatively treated patients stratified by Type D personality**. (C)** Mean VAS arm pain scores over time among surgically treated patients stratified by Type D personality. Values are presented as mean ± SD.

In patients without a Type D personality, mean VAS arm pain scores decreased from 57.89 ± 27 at baseline to 27.39 ± 26 after 6 weeks, and further to 15.30 ± 22 after 1 year while remaining stable at 17.73 ± 23 after 2 years. In patients with a Type D personality, mean arm pain scores decreased from 53.95 ± 26 at baseline to 22.32 ± 26 after 6 weeks, and further to 17.03 ± 24 after 1 year, but increased to 28.37 ± 32 at 2-year follow-up (Table 2; Figure 1A). Adjusted linear mixed-effects models demonstrated no significant differences in arm pain scores between patients with and without a Type D personality during the first 6 weeks nor over the first year of follow-up. However, over a 2-year period, patients with a Type D personality reported, on average, 11.72 points higher arm pain scores compared with patients without a Type D personality (*p* = 0.030) in the whole cohort (Table 3). Stratified analyses over the 2-year follow-up period showed that, within the conservative treatment group, patients with a Type D personality reported on average 17.41 points higher arm pain scores than patients without a Type D personality (*p* = 0.054, Figure 1B). Within the surgical treatment group, the corresponding mean difference was 9.30 points (*p* = 0.170, Figure 1C).

**Table 2:** Mean clinical outcome scores over time (mean ± SD) stratified by Type D personality and treatment type.

Clinical outcome Mean ±SD	Time Weeks	No Type D (*n* = 101)	No Type D, conservative (*n* = 38)	No Type D, surgical (*n* = 63)	Type D (*n* = 40)	Type D, conservative (*n* = 15)	Type D, surgical (*n* = 25)
VAS arm pain	Baseline	57.89 ± 27	47.89 ± 28	63.92 ± 25	53.95 ± 26	54.80 ± 27	53.44 ± 26
VAS arm pain	W6	27.39 ± 26	27.33 ± 27	27.43 ± 26	22.32 ± 26	15.25 ± 21	26.79 ± 29
VAS arm pain	W12	25.63 ± 29	24.17 ± 31	26.71 ± 28	21.33 ± 27	11.67 ± 16	29.07 ± 23
VAS arm pain	W26	17.91 ± 26	14.81 ± 27	19.90 ± 26	25.70 ± 29	21.18 ± 33	29.83 ± 25
VAS arm pain	W38	17.96 ± 23	12.40 ± 22	21.26 ± 24	18.42 ± 29	14.91 ± 30	23.25 ± 27
VAS arm pain	W52	15.30 ± 22	13.84 ± 21	16.22 ± 23	17.03 ± 24	13.75 ± 22	19.11 ± 25
VAS arm pain	W104	17.73 ± 23	18.52 ± 22	17.21 ± 24	28.37 ± 32	35.56 ± 25	23.78 ± 26
EQ-VAS	Baseline	54.63 ± 23	55.08 ± 22	54.37 ± 23	45.73 ± 21	46.60 ± 25	45.20 ± 19
EQ-VAS	W6	71.58 ± 20	65.41 ± 22	74.98 ± 17	67.77 ± 25	63.91 ± 31	70.00 ± 21
EQ-VAS	W12	70.84 ± 21	69.13 ± 24	72.13 ± 18	64.11 ± 24	62.33 ± 24	65.44 ± 26
EQ-VAS	W26	71.03 ± 23	68.00 ± 28	72.98 ± 20	66.96 ± 21	63.18 ± 24	70.42 ± 18
EQ-VAS	W38	67.20 ± 24	63.25 ± 27	69.45 ± 23	61.32 ± 25	58.00 ± 28	65.88 ± 20
EQ-VAS	W52	73.21 ± 21	70.32 ± 25	74.86 ± 20	64.48 ± 28	56.83 ± 31	71.54 ± 24
EQ-VAS	W104	70.04 ± 23	68.45 ± 24	71.09 ± 23	64.64 ± 20	58.00 ± 22	68.43 ± 18
VAS neck pain	Baseline	49.61 ± 27	43.92 ± 28	53.05 ± 26	60.97 ± 27	57.60 ± 28	63.00 ± 26
VAS neck pain	W6	32.28 ± 26	34.07 ± 29	31.26 ± 25	36.71 ± 26	32.00 ± 26	39.68 ± 26
VAS neck pain	W12	31.41 ± 28	25.66 ± 27	35.58 ± 29	33.07 ± 29	33.92 ± 27	32.44 ± 31
VAS neck pain	W26	23.84 ± 24	21.42 ± 24	23.33 ±24	31.65 ± 27	28.36 ± 31	34.67 ± 24
VAS neck pain	W38	25.50 ± 25	24.04 ± 26	26.39 ± 25	25.11 ± 28	19.09 ± 28	33.37 ±28
VAS neck pain	W52	21.56 ± 25	19.86 ± 26	22.54 ± 25	22.10 ± 23	26.17 ± 28	19.24 ± 19
VAS neck pain	W104	22.37 ± 24	28.03 ± 28	18.80 ± 20	26.03 ± 29	31.27 ± 28	16.72 ± 17
NDI	Baseline	34.14 ± 14	30.26 ± 14	36.56 ± 13	39.47 ± 16	39.73 ± 18	39.32 ± 15
NDI	W6	-	-	-	-	-	-
NDI	W12	25.71 ± 19	24.20 ± 23	26.87 ± 16	29.54 ± 20	24.00 ± 19	33.60 ± 20
NDI	W26	19.58 ± 19	20.56 ± 23	18.98 ± 16	28.61 ± 22	29.64 ± 27	27.67 ± 18
NDI	W38	20.52 ± 18	22.00 ± 22	19.66 ± 15	21.47 ± 21	22.73 ± 26	19.75 ± 11
NDI	W52	17.29 ± 17	18.58 ± 20	16.56 ± 15	21.41 ± 18	23.64 ± 23	19.88 ± 15
NDI	W104	16.47 ± 17	18.63 ± 20	15.07 ± 14	23.15 ± 18	26.60 ± 20	21.12 ± 16

Values are presented for VAS arm pain, EQ-VAS, VAS neck pain, and Neck Disability Index (NDI) at each time point (weeks). Groups are stratified into No Type D (total *n* = 101; conservative *n* = 38; surgical *n* = 63) and Type D personality (total *n* = 40; conservative *n* = 15; surgical *n* = 25), with outcomes further separated by conservative and surgical treatment subgroups.

**Table 3:** Adjusted linear mixed-effects models examining the association between Type D personality and clinical outcomes.

Clinical outcome	Time	β	95% CI	*p*	β	95% CI	*p*	β	95% CI	*p*
		A: Whole group			B: Conservative			C: Surgical		
**VAS Arm**	W6	−1.43	−11.84; 8.98	0.787	−14.24	−30.94; 2.47	0.094	6.17	−7.20; 19.54	0.363
**VAS Arm**	W52	3.93	−6.29; 14.14	0.450	−0.71	−17.20; 15.78	0.932	6.52	−6.57; 19.61	0.327
**VAS Arm**	W104	11.72	1.14; 22.30	0.030	17.41	−0.32; 35.13	0.054	9.30	−4.02; 22.61	0.170
**EQ-VAS**	W6	−4.96	−14.16; 4.28	0.290	0.00	−16.91; 16.91	1.000	−6.50	−17.33; 4.33	0.238
**EQ-VAS**	W52	−10.51	−20.06; 0.97	0.031	−11.29	−28.03; 5.45	0.183	−7.35	−19.25; 4.455	0.225
**EQ-VAS**	W104	−7.34	−17.17; 2.47	0.143	−11.63	−29.52; 6.25	0.200	−3.40	−15.06; 8.26	0.566
**VAS Neck**	W6	2.84	−7.88; 13.55	0.603	−4.94	−22.63; 12.76	0.582	8.47	−4.85; 21.79	0.211
**VAS Neck**	W52	−1.89	−12.6; 8.82	0.728	1.18	−16.49; 18.84	0.895	−3.78	−17.11; 9.56	0.577
**VAS Neck**	W104	1.08	−9.62; 11.77	0.843	7.12	−10.73; 24.96	0.432	−2.46	−15.65; 10.72	0.713
**NDI**	W12^*^	1.23	−5.93; 8.38	0.735	−6.52	−17.55; 4.48	0.241	5.07	−3.95; 14.09	0.268
**NDI**	W52	1.92	−5.08; 8.91	0.590	−2.12	−13.18; 8.94	0.704	2.84	−5.85; 11.53	0.518
**NDI**	W104	7.63	0.61; 14.65	0.033	6.03	−5.12; 17.17	0.285	7.36	−1.34; 16.06	0.096

^*^NDI was not collected at the 6-week follow-up; therefore, the 12-weeks time point is presented instead. All models are adjusted for clinical outcome baseline differences, age, gender, BMI. Model A compares patients with and without a Type D personality in the total study population. Model B compares conservatively treated patients with and without a Type D personality. Model C compares surgically treated patients with and without a Type D personality. All models were adjusted for baseline clinical outcome scores, BMI, age and gender.

### 3.3 Effects of Type D personality on EQ-VAS

**Figure 2:**
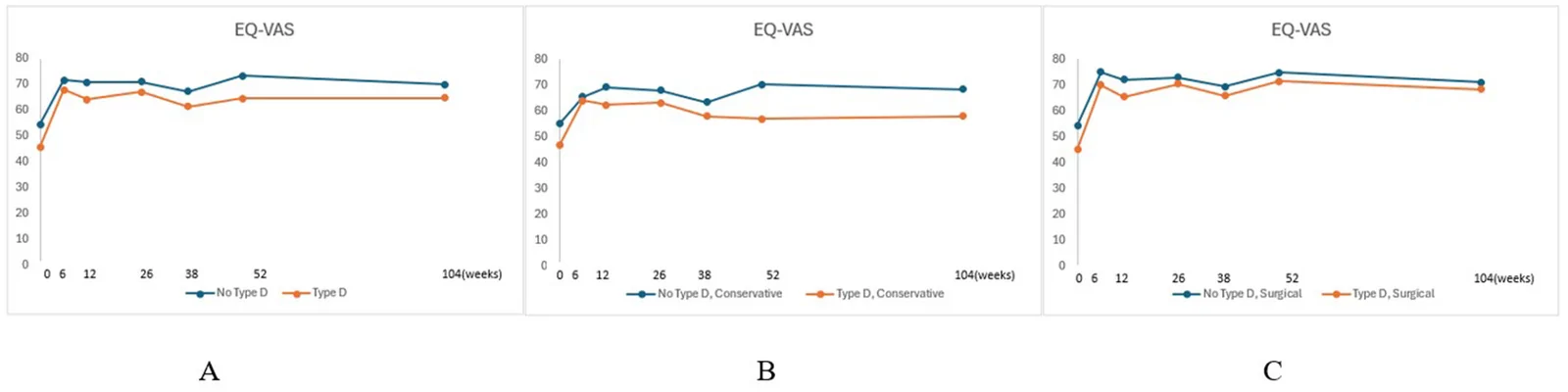
Clinical outcome trajectories for EQ-VAS over time stratified by Type D personality**. (A)** Mean EQ-VAS scores over time for the total study population comparing patients with and without a Type D personality**. (B)** Mean EQ-VAS scores over time among conservatively treated patients stratified by Type D personality. **(C)** Mean EQ-VAS scores over time among surgically treated patients stratified by Type D personality. Values are presented as mean ± SD.

In patients without a Type D personality, mean EQ-VAS scores increased from 54.63 ± 23 at baseline to 71.58 ± 20 at 6 weeks, followed by a further increase to 73.21 ± 21 at 1 year. Scores remained relatively stable at 70.04 ± 23 after 2 years. In patients with a Type D personality, mean EQ-VAS scores increased from 45.73 ± 21 at baseline to 67.77 ± 25 at 6 weeks, followed by a slight decrease to 64.48 ± 28 after 1 year, and remained stable at 64.64 ± 20 after 2 years (Table 2; Figure 2A). Adjusted linear mixed-effects models demonstrated no significant differences on the short term (β = −4.96, p=0.290) (Table 3). After 1 year however, patients with a Type D personality reported 10.51 lower EQ-VAS scores and thus worse overall perceived health compared with patients without a Type D personality, reaching a significant difference (p=0.031). Stratified analyses demonstrated that within the conservative treatment group, patients with a Type D personality reported on average 11.29 points lower EQ-VAS scores than patients without a Type D personality (*p* = 0.183, Figure 2B). Within the surgical treatment group, the corresponding mean difference was 7.35 points (*p* = 0.225, Figure 2C), in favor of patients without a Type D personality.

### 3.4 Effects of Type D personality on VAS neck pain

**Figure 3:**
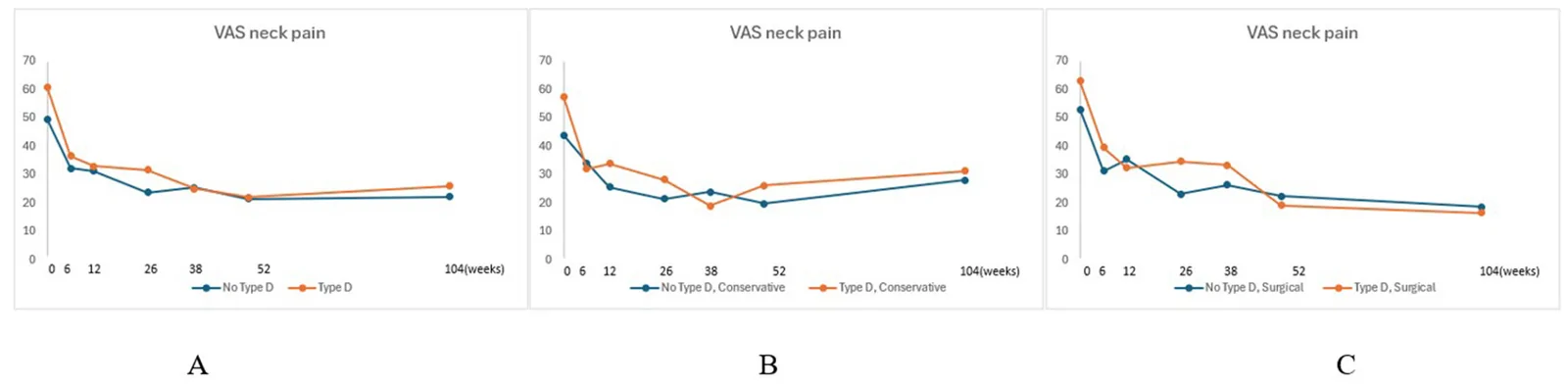
Clinical outcome trajectories for VAS neck pain over time stratified by Type D personality. **(A)** Mean VAS neck pain scores over time for the total study population comparing patients with and without a Type D personality. **(B)** Mean VAS neck pain scores over time among conservatively treated patients stratified by Type D personality. **(C)** Mean VAS neck pain scores over time among surgically treated patients stratified by Type D personality. Values are presented as mean ± SD.

In patients without a Type D personality, mean VAS neck pain scores decreased from 49.61 ± 27 at baseline to 32.28 ± 26 at 6 weeks, followed by a further decrease to 21.56 ± 25 after 1 year. Scores remained stable at 22.37 ± 24 after 2 years. Similarly, in patients with a Type D personality, scores decreased from 60.97 ± 27 at baseline to 36.71 ± 26 at 6 weeks, 22.10 ± 23 at 1 year, and 26.03 ± 29 after 2 years (Table 2; Figure 3A). Adjusted linear mixed-effects models showed no significant differences in neck pain between groups, either during the first 6 weeks, the first year of follow-up or the 2-year period (Table 3, Figure 3B, C).

### 3.5 Effects of Type D personality on NDI

**Figure 4:**
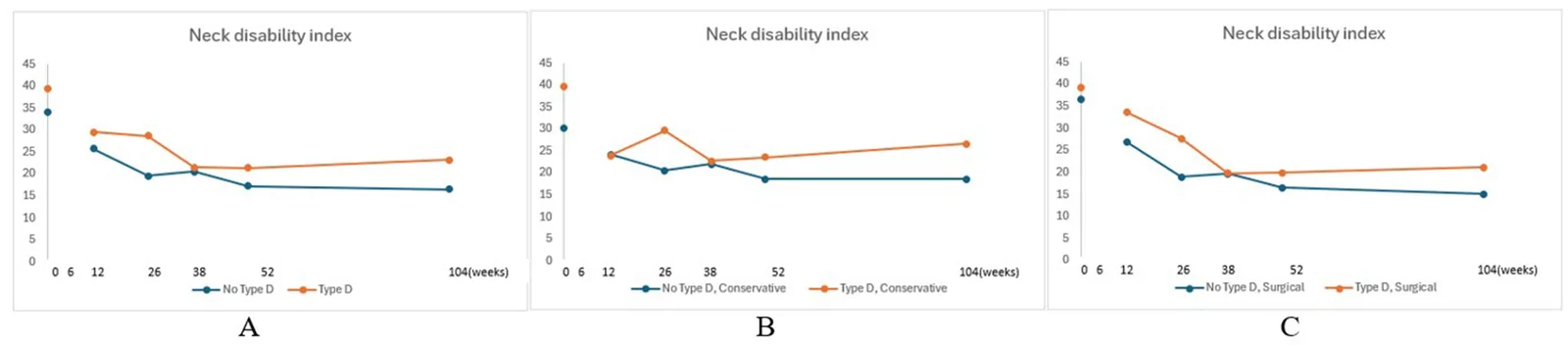
Clinical outcome trajectories for Neck Disability Index (NDI) scores over time stratified by Type D personality. (**A)** Mean NDI scores over time for the total study population comparing patients with and without a Type D personality. **(B)** Mean NDI scores over time among conservatively treated patients stratified by Type D personality. **(C)** Mean NDI scores over time among surgically treated patients stratified by Type D personality. Values are presented as mean ± SD.

In patients without a Type D personality, mean NDI decreased from 34.14 ± 14 at baseline to 17.29 ± 17 after 1 year and 16.47 ± 17 after 2 years. In patients with a Type D personality, mean NDI scores decreased from 39.47 ± 16 at baseline to 21.41 ± 18 at 1 year and 23.15 ± 18 after 2 years, respectively (Table 2; Figure 4A). Adjusted linear mixed-effects models indicated no significant differences in NDI scores between patients with and without a Type D personality during short-term follow-up or 1 year. However, over the 2-year follow-up period, patients with a Type D personality reported, on average, 7.63 points higher NDI scores compared with patients without a Type D personality (*p* = 0.033) (Table 3). Stratified analyses demonstrated that within the conservative subgroup, patients with a Type D personality reported 6.03 points higher NDI scores than those without a type d personality (*p* = 0.285), which was 7.36 points in the surgical treatment subgroup (*p* = 0.096) (Figure 4B, C).

### 3.6 Association of Type D personality with demographic factors

Baseline factors associated with the presence of a Type D personality were explored using independent *t*-tests for continuous variables and chi-square and/or Fisher's exact tests for categorical variables (Appendix A). Patients with a Type D personality reported a longer time since their first general practitioner visit compared with patients without a Type D personality (mean difference 8.22 months, *p* = 0.018), although the total duration of complaints did not differ significantly between groups (mean difference 2.46 months, *p* = 0.296). Furthermore, marital status was associated with Type D personality, with a lower proportion of patients with a Type D personality being married or living with a partner compared with patients without a Type D personality (33% vs. 16%, *p* = 0.038).

In addition, patients with a Type D personality reported lower baseline EQ-VAS scores, reflecting lower overall perceived health, compared with patients without a Type D personality (mean difference −8.91 points, *p* = 0.034). In addition, patients with a Type D personality reported higher baseline VAS neck pain scores (mean difference 11.36 points, *p* = 0.025). No significant differences were observed in baseline VAS arm pain scores between patients with and without a Type D personality. No other significant associations were identified among the demographic, clinical, lifestyle, medication-related, or treatment-related factors assessed (Appendix A).

## 4 Discussion

The present study aimed to evaluate the association between Type D personality and clinical outcomes following conservative or surgical treatment for cervical radiculopathy, and to determine whether these associations differed between treatment strategies. No significant differences in recovery were observed during the short-term follow-up period across any of the assessed outcomes. However, over the long-term follow-up period, differences emerged for several patient-reported outcomes, with patients with a Type D personality demonstrating less favorable long-term recovery. These findings suggest that Type D personality may be a relevant factor to consider during patient counseling and long-term expectation management.

### 4.1 Interpretation of findings

The association between Type D personality and arm pain was not evident during the first year of follow-up but became apparent at 24 months. This suggests that psychological vulnerability associated with Type D personality may be more closely related to long-term adaptation, symptom persistence, and perception of recovery rather than the initial response to treatment. Although the association was significant in the overall cohort (β = 11.72, *p* = 0.030), similar numerical differences were observed within both treatment subgroups but did not reach statistical significance (conservative treatment: β = 17.41, *p* = 0.054; surgical treatment: β = 9.30, *p* = 0.170). This may be explained by the smaller sample sizes within the subgroup analyses, resulting in reduced statistical power.

Interestingly, patients with a Type D personality reported lower baseline EQ-VAS scores, reflecting lower self-perceived overall health, compared with patients without a Type D personality (β = −8.91, *p* = 0.034). Moreover, this association became stronger during follow-up, with patients with a Type D personality reporting significantly lower EQ-VAS scores after 1 year (β = −10.51, *p* = 0.031). Although the mean difference appeared somewhat larger among conservatively treated patients (β = −11.29, *p* = 0.183) compared with surgically treated patients (β = −7.35, *p* = 0.225), neither subgroup reached statistical significance, again potentially due to limited statistical power. The association between Type D personality and poorer self-perceived health supports the concept that psychological factors influence recovery beyond pain intensity alone and contribute to the broader perception of health and wellbeing. Recovery from cervical radiculopathy should therefore be considered a multidimensional process, in which psychological wellbeing may play an important role. At 2 years, the difference in EQ-VAS between groups appeared to decrease, which may reflect the influence of additional factors over longer follow-up periods that contribute to overall perceived health.

No significant association was observed between Type D personality and neck pain. Patients with a Type D personality even reported higher baseline neck pain scores compared with patients without a Type D personality (β = 11.36, *p* = 0.025). The absence of a similar association for neck pain compared with arm pain may reflect differences in the clinical presentation and perception of these symptoms. Although both arm and neck pain in cervical radiculopathy are influenced by a combination of biological and psychosocial factors, arm pain is often the predominant symptom driving disability and treatment decisions in this patient population. Psychological factors may influence how patients perceive, cope with, and adapt to persistent symptoms, which could contribute to the observed association with long-term arm pain and disability. However, the lack of association with neck pain suggests that the relationship between Type D personality and pain outcomes may differ across symptom domains and requires further investigation.

A similar pattern as to arm pain was observed for disability measured with the NDI, with differences between patients with and without a Type D personality becoming more apparent over longer follow-up. Although short-term disability improvement was comparable between groups, patients with a Type D personality reported higher disability scores after 2 years.

Beyond the association with clinical outcomes, we examined potential baseline factors associated with the presence of a Type D personality. The interval between symptom onset and the first general practitioner visit was longer among patients with a Type D personality compared with those without a Type D personality (β = 8.22, *p* = 0.018), whereas the overall duration of complaints did not differ between groups. This may indicate that patients with a Type D personality seek medical attention later after symptom onset. One possible explanation is that individuals with a Type D personality may experience lower symptom-related distress or increased symptom awareness, this is interesting given that previous studies have demonstrated higher levels of psychological and somatic distress among these individuals (de Fruyt and Denollet, 2002). However, when examining the individual components of the Type D personality construct, one of its characteristics is that individuals with a Type D personality often experience difficulty expressing their emotions. This may also extend to communicating their physical symptoms, potentially causing them to delay seeking medical attention. Another baseline characteristic associated with Type D personality was marital status. Patients with a Type D personality were more frequently living alone or not married compared with patients without a Type D personality. This observation aligns with previous research showing that individuals with a Type D personality often report lower levels of perceived social support across different populations (Dehghani, 2018; O'Riordan and Costello, 2026; Xie et al., 2026).

### 4.2 Comparison with existing literature

Although Type D personality has been extensively studied in cardiovascular populations, evidence regarding its relevance for clinical outcomes in patients with spinal disorders remains limited (Riediger et al., 2026, 2025; Eroglu et al., 2022; Lodder et al., 2023; Izquierdo Coronel et al., 2024; Kupper and Denollet, 2018; Al Nakhebi et al., 2025). The present study is the first to investigate the association between Type D personality and (both short and long-term) clinical outcomes in patients with cervical radiculopathy.

Riediger et al. evaluated the association between Type D personality and clinical outcomes in a cross-sectional cohort of 300 patients presenting to a university spine outpatient clinic and reported higher VAS pain scores among patients with a Type D personality (Riediger et al., 2026). However, the specific pain location (arm or neck pain) was not reported, limiting direct comparison with our findings. In a subsequent prospective cohort of 200 patients undergoing elective spine surgery, Riediger et al. found that patients with a Type D personality reported higher pain scores and lower treatment satisfaction at 12 months (Eroglu et al., 2022). These findings are consistent with our observation that Type D personality was associated with less favorable long-term patient-reported outcomes, including higher arm pain and lower overall perceived health. In contrast, Eroglu et al. found no association between Type D personality and pain intensity in patients with spinal cord injury (Riediger et al., 2025). However, this population differs substantially from patients with cervical radiculopathy, making comparisons difficult.

Given that Type D personality has been associated with higher levels of depressive symptoms and psychological distress (Denollet et al., 2021; van Dooren et al., 2016), previous research on these related psychological constructs may provide relevant context for interpreting our findings. Although Type D personality itself has not yet been studied in patients with cervical radiculopathy, several studies have demonstrated an association between depressive symptoms or psychological distress and clinical outcomes in this population (Cunningham et al., 2025; Goedmakers et al., 2022; Skeppholm et al., 2017) In line with our results, Goedmakers et al. found that persistent anxiety or depressive symptoms were associated with worse NDI scores after anterior cervical discectomy (ACDF) at 2 years follow-up (Goedmakers et al., 2022). Similarly, Skeppholm et al. reported an association between preoperative psychological distress and NDI scores at 2 year follow-up (Skeppholm et al., 2017). Cunningham et al. further demonstrated that preoperative depression and severe psychological symptomatology increased the risk of unfavorable trajectories for disability, neck pain, and arm pain following anterior cervical discectomy and fusion (Al Nakhebi et al., 2025). Together, these findings support the relevance of psychological factors in long-term recovery after cervical radiculopathy treatment and may partly explain the less favorable long-term outcomes observed among patients with a Type D personality in our cohort.

### 4.3 Clinical implications

Several mechanisms may explain the observed associations between Type D personality and long-term outcomes. Patients with a Type D personality may experience greater symptom perception, less adaptive coping strategies, and difficulties in adapting to persistent symptoms, potentially affecting long-term recovery. Identifying patients with increased psychological vulnerability may therefore be relevant for personalized counseling and expectation management.

Psychological interventions may represent a potential strategy to improve outcomes. Jin et al. demonstrated in a randomized controlled trial among patients undergoing ACDF that preoperative communication combined with structured psychological support resulted in lower postoperative pain and clinically relevant improvement in NDI at 1 month follow-up (Jin et al., 2026). Whether such benefits persist over the longer term remains unknown. Future research should investigate whether psychological interventions tailored to specific psychological profiles, including Type D personality, can further optimize treatment outcomes and patient satisfaction in patients with cervical radiculopathy.

### 4.4 Limitations and strengths

The use of data from a prospective multi-center cohort with 2 years follow-up allowed for a comprehensive evaluation of the relationship between Type D personality and clinical outcomes in patients with cervical radiculopathy. The inclusion of both conservatively and surgically treated patients enhances the generalizability of our findings and provides a more complete representation of the clinical spectrum of this condition. This study also includes multiple limitations. the observational design of the study precludes causal inference, and the directionality of the association between Type D personality and clinical outcomes cannot be definitively established. In addition, performing subgroup analyses according to treatment strategy results in smaller sample sizes, which may reduce statistical power and increase the likelihood that clinically relevant differences do not reach statistical significance. Furthermore, treatment allocation in this observational cohort was not randomized and was based on clinical decision-making and shared decision-making between patients and physicians. Therefore, residual confounding by indication cannot be excluded.

## 5 Conclusion

In conclusion, this study demonstrates that higher baseline Type D personality is associated with less favorable long-term clinical outcomes in patients with cervical radiculopathy, particularly with respect to arm pain and overall health-related quality of life. These associations were not evident in the early phase of recovery but became significant over time, suggesting that Type D personality predominantly influences long-term adaptation and persistence of symptoms rather than short-term treatment response.

## Data Availability

The data supporting the findings of this study are available upon reasonable request. Access requests must be submitted to the Medical Research Ethics Committee Leiden-The Hague-Delft (METC LDD). Following ethical approval, anonymized data may be made available to qualified researchers.
