# Supplementary material for: The prevalence of Type D personality and its association with clinical outcomes in patients with cervical radiculopathy after either conservative or surgical treatment
Source: Front Hum Neurosci. 2026 Aug 20;20:1887901. doi: 10.3389/fnhum.2026.1887901 (PMC13538433; doi:10.3389/fnhum.2026.1887901)
Supplement: Supplementary file 1 [file Table_1.docx]

**Appendix A: Which baseline factors are associated with the presence of a Type D personality?**

- For all continuous variables an independent t-test was performed: age, BMI, length of complaints, months since first GP visit, baseline VAS arm pain, baseline EQ-VAS, baseline VAS neck pain, baseline NDI

- For all categorical variables chi-square and/or Fisher’s exact tests have been performed: gender, treatment, daytime activities, education, sick leave, alcohol consumption, smoking behaviour, family member, married/moved in, participation in sports, use of paracetamol/NSAID/tramadol/morphine

|  |  | **Type D personality** | | |
| --- | --- | --- | --- | --- |
|  | **B** | | **95% CI** | **p** |
| Age, years | -1.27 | | -2.34;4.87 | 0.489 |
| Gender |  | |  | 0.453 |
| Body Mass Index, kg/m2 | 0.51 | | -2.08;1.06 | 0.522 |
| Treatment |  | |  | 0.989 |
| Daytime Activities |  | |  | 0.578 |
| Education |  | |  | 0.341 |
| Sick leave |  | |  | 0.130 |
| Alcohol consumption |  | |  | 0.284 |
| Smoking behaviour |  | |  | 0.618 |
| Family member with CRS? |  | |  | 0.563 |
| Married and/or moved in |  | |  | 0.038 |
| Participation in sports |  | |  | 0.141 |
| Length of complaints  Months since first GP visit Arm pain compared to Neck pain | 2.46 8.22 | | -7.06;2.16 -15.00; -1.43 | 0.296 0.018 0.395 |
| Use of paracetamol |  | |  | 0.282 |
| Use of NSAID’s |  | |  | 0.803 |
| Use of tramadol |  | |  | 0.274 |
| Use of morphine |  | |  | 0.907 |
| Baseline VAS Arm | -3.94 | | -3.96;13.84 | 0.433 |
| Baseline EQ-VAS | -8.91 | | 0.70;17.12 | 0.034 |
| Baseline VAS Neck | 11.36 | | -21.24; -1.48 | 0.025 |
| Baseline NDI | 5.33 | | -11.07;0.40 | 0.068 |
